# Enhancing the security of horizontal canal BPPV repositioning maneuvers: insights from virtual simulation

**DOI:** 10.3389/fneur.2025.1560324

**Published:** 2025-05-16

**Authors:** Jianxin Li, Haoxiang Hu, Yihan Zhang, Xiaokai Yang

**Affiliations:** ^1^Department of Neurology, Wenzhou Third Clinical Institute Affiliated to Wenzhou Medical University, Wenzhou People’s Hospital, Wenzhou, China; ^2^School of Disaster and Emergency Medicine, Tianjin University, Tianjin, China; ^3^Department of Neurology, Postgraduate Training Base Alliance of Wenzhou Medical University (Wenzhou People’s Hospital), Wenzhou, China

**Keywords:** BPPV, virtual simulation, repositing maneuver, otolith, Zuma maneuver

## Abstract

Horizontal canal benign paroxysmal positional vertigo (hc-BPPV) presents distinct challenges in both diagnosis and treatment. This study aims to explore the trajectories of otoliths during different hc-BPPV reduction maneuvers using advanced virtual simulation technology, and evaluate the theoretical effectiveness and security of these maneuvers while providing recommendations for optimizing current techniques or developing innovative approaches. We created a high-fidelity, three-dimensional (3D) virtual model of the human vestibular system that accurately depicts the horizontal semicircular canal. Utilizing the Unity 3D platform combined with NVIDIA PhysX physics engines, and simulated various hc-BPPV repositioning maneuvers, including the Lempert roll, Gufoni maneuver, forced prolonged positioning, and Zuma maneuvering. The otoliths were modeled as spherical particles, with their motion trajectories analyzed through precise quaternion rotation. Then, we collected and examined the 3D trajectory, velocity, and positional changes of the otoliths in relation to key anatomical landmarks. The simulations indicate that traditional maneuvers such as Lempert and Gufoni are effective in repositioning otoliths, but along with the risk that otoliths mistakenly inserted into other semicircular canals. In contrast, Zuma maneuver is more complex to execute, but it provides higher security. The improved Zuma maneuver not only simplifies the procedure but also maintains high efficacy and security standards. This study provides a comprehensive comparison of various hc-BPPV repositioning maneuvers using virtual simulation technology. The modified Zuma maneuver is proposed as a more effective and safer treatment method. Further clinical validation and individual adjustment studies are necessary to optimize this approach and improve hc-BPPV management strategies.

## Highlights

- This paper provides a comprehensive comparison of various hc-BPPV repositioning maneuvers using virtual simulation technology.
- The comparison of various repositioning maneuvers for horizontal Canal BPPV.

## 1 Introduction

Benign Paroxysmal Positional Vertigo (BPPV) is one of the most prevalent vestibular disorders, affecting approximately 2.4% of the general population (1). Given the noteworthy prevalence of BPPV, its health care and societal impacts are tremendous. The remarkable feature of BPPV is that specific head movements cause short-term and intense vertigo attacks, which seriously affect the quality of life, especially for the elderly. These debilitating symptoms arise from the displacement of otoconia-small calcium carbonate crystals-from the otolith organs into the semicircular canals. Horizontal canal BPPV (hc-BPPV) accounts for 10–20% of all BPPV cases (2), which poses a unique challenge to diagnosis and treatment. The impact of BPPV on patients’ well-being is profound. Frequent and disabling vertigo attacks can lead to functional limitations, psychological distress, reduced mobility, and an increased risk of falls (3), and almost 86% of patients with BPPV will suffer some interrupted daily activities and lost days at work due to BPPV (1). Notably, hc-BPPV is particularly problematic as it is more easily provoked by changes in head position.

Over the past two decades, researchers have developed various repositioning maneuvers to treat hc-BPPV, including the Lempert roll (4), Gufoni maneuver (5), forced prolonged positioning (6), and Zuma maneuver (7). However, although they are widely used in clinic, compared with the curative effect of operations, they come to inconsistent conclusions (8–12). This inconsistency indicates the potential limitations of the current treatment scheme or the superiority of certain maneuvers over others (13). Moreover, the difficulty in determining the affected side in hc-BPPV cases further complicates the implementation of repositioning treatments (14). Virtual simulation technology has emerged as a powerful tool for in-depth analysis of BPPV repositioning maneuvers (15).

Recent studies have explored various aspects of these maneuvers, including:

1. The efficacy of repositioning maneuvers for otoconia in different positions (13).
2. Security considerations, such as the potential for inadvertent otoconia displacement from the utricle to the semicircular canals during repositioning (16).
3. Strategies to mitigate vertigo during the repositioning process (17).
4. The potential for self-administered repositioning treatments (18).
5. Effective methods for dislodging otoconia adhered to the cupula (19, 20).

To fill these knowledge gaps and enhance our understanding of the precise mechanisms of various hc-BPPV treatment strategies, this study utilized innovative virtual simulation technology to analyze the trajectories of otoconia during different repositioning procedures. Our primary research objectives are:

1. To utilize virtual simulation technology for precise analysis of otoconia trajectories during various hc-BPPV repositioning maneuvers.
2. To evaluate the theoretical efficacy of different maneuvers, including their otoconia clearance efficiency and potential risks of canal reentry.
3. To investigate the effectiveness of maneuvers in repositioning otoconia from different locations within the horizontal semicircular canal, including the long arm, ampulla, and short arm.
4. To propose recommendations for optimizing existing repositioning maneuvers or developing novel approaches based on virtual simulation results.

The primary purposes of this study are to provide a deeper theoretical foundation for hc-BPPV treatment, enabling clinicians to better understand the mechanisms of various repositioning maneuvers and offering scientific evidence for selecting the most appropriate treatment protocols. It not only contributes to improving the efficacy of existing treatments but also paves the way for developing more effective and precise repositioning maneuvers, ultimately enhancing the treatment outcomes and quality of life for hc-BPPV patients.

## 2 Materials and methods

### 2.1 Virtual model development

David (21) originally created the models of bone labyrinth and membranous labyrinth. Based on high-resolution micro-CT scan of human temporal bone, these models provide anatomical accurate data of maze structure. On this basis, we developed a complex 3D virtual model of the human vestibular system.

We extended this work by accurately locating the membrane labyrinth in the 3D space. This was achieved through a rigorous calibration process using a standard bone labyrinth model as a reference. This calibration ensured anatomical accuracy and consistency in the spatial relationships between the various vestibular structures. Based on this calibrated model, we constructed our virtual simulation model using Unity 3D (version 2020.3) integrated with the NVIDIA PhysX physics engine. This combination allows for accurate simulation of both rigid body dynamics and fluid interactions, crucial for modeling otoconia movement within the endolymph (16, 22, 23).

### 2.2 Simulation architecture

We implemented a browser-server architecture for the simulation platform. This design supports remote access to the simulation model, efficient distributed computing and data processing, and collaborative research, allowing multiple researchers to interact with the model, run simulations, and analyze results from various locations simultaneously.

### 2.3 Otoconia and fluid dynamics modeling

Otoconia were modeled as spherical particles with properties matching those of human otoconia: radius ranging from 0.5 to 15 nanometers (nm), with an average of 7.5 nm, and density of 2.71 g/cm^3^ (calcium carbonate composition) (24–26). The endolymph density was set at 1 g/cm^3^. We calculated the buoyancy force at 3.62 m/s^2^, considering the density differential between otolith and endolymph, and incorporated advanced modeling of fluid resistance and viscosity effects for realistic otoconia movement simulation.

### 2.4 Repositioning maneuver simulation

We simulated various repositioning maneuvers for horizontal canal BPPV (hc-BPPV), categorized into two main groups:

1. Supine position maneuvers: Baloh’s method and its variations, Lempert (Barbecue) roll and its modifications, Tirelli’s modified Baloh method, Kim’s Cupulolith Repositioning Maneuver (CuRM), and Zuma’s method and its variations.
2. Non-supine position maneuvers: Gufoni maneuver and its variations, Appiani maneuver, Li’s quick roll method, and Forced Prolonged Position (FPP) therapy.

Each maneuver was accurately reproduced in the virtual environment, with head positions accurately timed and positioned according to standard clinical protocols.

### 2.5 Simulation scenarios

In order to comprehensively evaluate the efficacy and potential risks of each maneuver, we simulated various scenarios and considered different otolith starting positions: long arm of the horizontal canal, short arm of the horizontal canal, near the ampulla (in the ampulla), cupula (for cupulolithiasis simulation), and utricle (to assess risk of canal entry during maneuvers).

### 2.6 Data collection and analysis

For each simulation scenario and maneuver, we collected and analyzed 3D trajectories of individual otoconia, time-stamped positions relative to key anatomical landmarks, and velocity and acceleration profiles of otoconia. We implemented quaternion rotations to simulate head movements and positional changes, allowing for precise control and analysis of complex 3D movements. Our model supports both world and object coordinate systems, enabling comprehensive analysis of otoconia behavior relative to different reference frames.

### 2.7 Evaluation criteria

We established the following criteria to evaluate each maneuver:

1. Efficacy: Ability to reposition otoconia from different starting locations within the semicircular canal.
2. Security: Risk of otoconia entering the semicircular canal from the utricle during the maneuver.

Virtual simulation videos were created for each repositioning maneuver to facilitate detailed observation and analysis of otoconia trajectories.

## 3 Results

### 3.1 Efficacy in otolith repositioning

Our comprehensive analysis of various repositioning maneuvers for Horizontal Canal Benign Paroxysmal Positional Vertigo (HC-BPPV) reveals significant differences in their effectiveness and potential risks.

For detailed visualization of otolith trajectories during each maneuver, refer to the virtual simulation videos provided in the Supplementary materials.

1. Long arm: most maneuvers (9 out of 12) effectively reposition otoliths in the long arm of the horizontal canal. The exceptions are Appiani’s maneuver and the Forced Prolonged Position (FPP) on the affected side.
2. Short arm: 10 out of 12 maneuvers successfully reposition otoliths in the short arm. Again, Appiani’s maneuver and FPP on the affected side are the exceptions.
3. Ampulla: only 5 maneuvers (Tirelli Modified Lempert, Kim’s CuRM, Zuma, Vats Modified Zuma, and Li Quick Roll) effectively address otoliths in the ampulla. This highlights the challenge in treating ampullary otoliths.

### 3.2 Risk of otolith entry into canal

7 out of 12 maneuvers carry a risk of otoliths entering the semicircular canal during the procedure. This risk is notably absent in Gufoni’s maneuver, Li Quick Roll, and both FPP techniques, potentially making these safer options in certain clinical scenarios.

### 3.3 Comprehensive approaches

Lempert modified by Tirelli, CuRM modified by Kim, Zuma modified by Vats are the most comprehensive reduction methods at present. They effectively reposition otoliths in all areas of the canal (long arm, short arm, and ampulla). However, they also carry the risk of otolith entry into the canal, necessitating careful consideration in their application.

Gufoni’s maneuver has the following advantages and disadvantages in application:

1. Single-side application: effectively repositions otoliths in the posterior arm of the long arm and the short arm of the horizontal canal without risking canal entry. However, it’s ineffective for otoliths in the ampulla of the long arm.
2. Bilateral application: remarkably, when performed bilaterally, the Gufoni maneuver can reposition otoliths in any position of the bilateral lateral semicircular canals. This includes otoliths in the ampulla of the long arm, which cannot be repositioned by a single-side maneuver.
3. Security profile: maintains a low risk of otolith entry into the canal during the procedure.
4. Versatility: the ability to address otoliths in various positions through bilateral application makes it a versatile option in clinical practice.

FPP techniques show limited effectiveness but may be valuable in specific cases due to their low risk of canal entry. Li Quick Roll offers a balance of effectiveness and safety, addressing all canal areas without the risk of canal entry.

**Figure 1:**
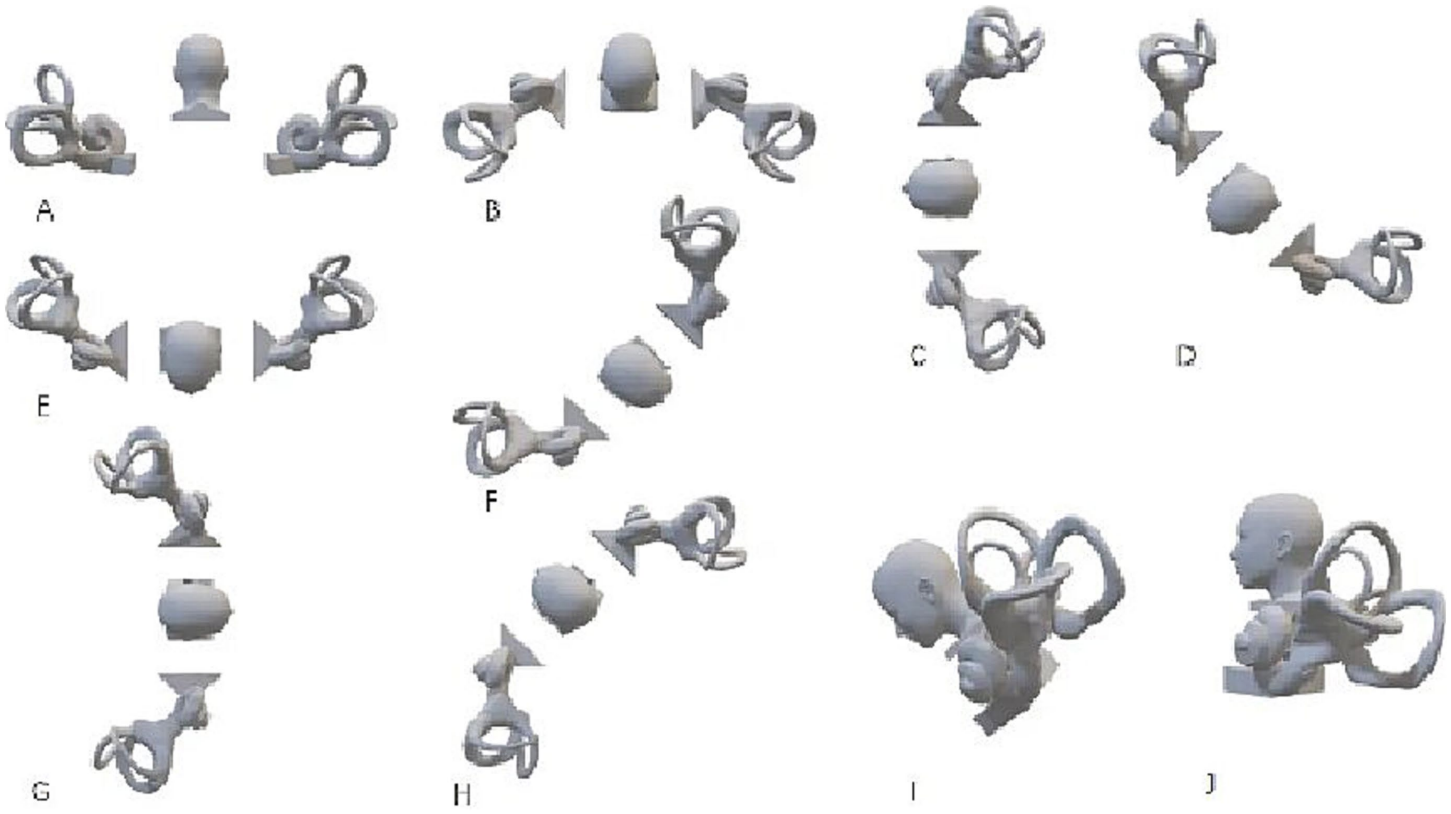
Head positions for horizontal canal repositioning maneuvers. **(A)** Upright sitting position, facing forward, Quaternion: +1.000 + 0.000i +0.000j +0.000 k. **(B)** Supine position with head elevated 75 degrees, Quaternion: +0.793 + 0.609i +0.000j +0.000 k. **(C)** Left lateral recumbent position, Quaternion: +0.500 + 0.500i −0.500j +0.500 k. **(D)** Left lateral recumbent position, nose turned 45 degrees downward, Quaternion: +0.271 + 0.271i −0.653j +0.653 k. **(E)** Prone position (face down), Quaternion: +0.000 + 0.000i −0.707j +0.707 k. **(F)** Right lateral recumbent position, nose turned 45 degrees downward, Quaternion: −0.271 −0.271i −0.653j +0.653 k. **(G)** Right lateral recumbent position, Quaternion: −0.500 −0.500i −0.500j +0.500 k. **(H)** Supine position with head turned 45 degrees to the left, Quaternion: +0.653 + 0.653i −0.271j +0.271 k. **(I)** Sitting position with head bent forward 45 degrees, facing left, Quaternion: +0.653–0.271i −0.271j +0.653 k. **(J)** Upright sitting position, facing left, Quaternion: +0.707 + 0.000i +0.000j +0.707 k.

The Table 1 outlines the steps for major repositioning maneuvers (for left horizontal canal BPPV unless otherwise specified). Letters represent different head positions as shown in Figure 1. For most maneuvers, each position is typically held for 30 s or until nystagmus subsides. Exceptions include:

1. Gufoni maneuver: each position is held for 1–2 min.
2. Zuma maneuver: each position is held for 3 min.
3. Forced Prolonged Position (FPP): patients maintain the final position for 12 h.

**Table 1:** Comparison of repositioning maneuvers for horizontal canal BPPV.

Maneuver	Steps	Long arm	Short arm	Ampulla	Otolith entry to canal	Reference	Video Link
Baloh’s Original (180 roll)	B → G → E	Yes	Yes	No	Possible	Baloh et al. (27)	Video1
vf	B → G → E → C	Yes	Yes	No	Possible	Lempert and Tiel Wilk (4)	Video2
Baloh Modified (360 roll)	B → G → E → C → B	Yes	Yes	No	Possible	Baloh et al. (27)	Video3
Tirelli Modified Lempert	B → C → B → G → E → C → J	Yes	Yes	Yes	Possible	Tirelli et al. (28)	Video4
Kim’s CuRM	B → C → D → B → G → E → C → J	Yes	Yes	Yes	Possible	Kim et al. (8)	Video5
Zuma	G → B → C → I → J	Yes	Yes	Yes	Possible	Zuma e Maia et al. (7)	Video6
Vats Modified Zuma	G → B → C → D → I → J	Yes	Yes	Yes	Possible	Rajguru et al. (26)	Video7
Gofoni (right BPPV)	J → C → D → J	Yes	Yes	No	No	Gufoni et al. (5)	Video8
Appiani (right BPPV)	J → C → H → J	No	No	No	Possible	Appiani et al. (33)	Video9
Li Quick Roll (right BPPV)	G → C → J	Yes	Yes	Yes	No	Li et al. (30)	Video10
FPP-Healthy-side	J → C → J	Yes	Yes	No	No	Vannucchi et al. (6)	Video11
FPP-Affected side	J → C → J	No	No	No	No	Chiou et al. (34)	Video11

## 4 Discussion

This study utilized virtual simulation technology to systematically analyze and compare various repositioning methods for horizontal semicircular canal benign paroxysmal positional vertigo (HSC-BPPV). Our aim was to evaluate the effectiveness of these methods, understand their mechanisms of action, and provide recommendations for optimal repositioning strategies in clinical practice.

The development of repositioning maneuvers involving a supine position reflects the evolving understanding of HSC-BPPV treatment. From Baloh et al. (27) initial 180° roll, to Lempert (4) 270° roll, and Baloh’s improved 360° roll, these maneuvers have been continuously optimized. Tirelli (28) and Kim et al. (8) further refined these methods, incorporating affected-side lying positions and larger head rotation angles.

Virtual simulation results show that these methods are effective in repositioning otoliths in both the long and short arms of the horizontal semicircular canal. However, they generally share a common issue: the supine position may cause otoliths from the utricle to enter the semicircular canal (16). Additionally, some methods (such as Baloh’s original method, Lempert 270° roll maneuver) are less effective in repositioning otoliths in the ampulla (13). Maneuvers that avoid the supine position, such as the Gufoni maneuver (5) and Appiani maneuver (29), offer alternatives that avoid the supine position. These methods typically start from a sitting position and achieve repositioning through rapid side-lying. Li’s (30) method and Vannucchi et al. (6) Forced Prolonged Position (FPP) also fall into this category.

Virtual simulations demonstrate that these methods have advantages in preventing otoliths from entering the semicircular canal (31). The Gufoni maneuver, in particularly, not only has the shortest repositioning path but can also reposition otoliths in any location through bilateral manipulation (32).

Our analysis reveals the importance of two key head positions in HSC-BPPV repositioning:

1. Affected-side lying: Moves otoliths (including canalith and cupulolith) away from the ampulla on the long arm side.
2. Healthy-side lying: Facilitates the entry of otoliths from both long and short arms into the utricle.

Notably, the supine position is the main cause of otoliths entering the semicircular canal and should be avoided when possible (16).

The comparison of various repositioning maneuvers for horizontal canal BPPV reveals distinct advantages and disadvantages for each approach (Table 2). Maneuvers involving supine positions, such as the Lempert roll and Baloh’s method, are effective for repositioning otoliths in the long arm posterior and short arm of the horizontal semicircular canal. However, these maneuvers carry the risk of otoliths entering the semicircular canal and involve a long otolith movement path, which may increase patient discomfort.

**Table 2:** Comparison of various repositioning maneuvers for horizontal canal BPPV.

Maneuver	Advantages	Disadvantages
Maneuvers involving supine position	Effective for otoliths in long arm posterior and short arm	Risk of otoliths entering semicircular canal; Long otolith movement path
Gufoni maneuver	Short repositioning path; No risk of otoliths entering semicircular canal; Can be performed bilaterally	Cannot directly reposition otoliths in ampulla of the long arm side
Appiani maneuver	Targets apogeotropic HSC-BPPV	Upward head turn may cause otoliths from utricle to enter semicircular canal
FPP	Principle similar to Gufoni maneuver	Prolonged lying position lacks solid evidence

The Gufoni maneuver presents several advantages, including a short repositioning path and no risk of otoliths entering the semicircular canal. Additionally, it can be performed bilaterally, enhancing its versatility. However, this maneuver cannot directly reposition otoliths in the ampulla of the long arm side, which may limit its effectiveness in certain cases.

The Appiani maneuver is specifically designed to target apogeotropic HSC-BPPV. While this specificity can be advantageous, the upward head turn involved in the maneuver may cause otoliths from the utricle to enter the semicircular canal, potentially exacerbating the condition.

Forced Prolonged Position (FPP) therapy operates on a principle similar to the Gufoni maneuver. However, the prolonged lying position required in this approach lacks solid evidence to support its efficacy, which may limit its clinical application.

These findings underscore the importance of carefully selecting the appropriate repositioning maneuver based on the specific characteristics of each BPPV case, considering factors such as the location of otoliths, patient comfort, and potential risks associated with each technique.

Based on our analysis, we recommend the following repositioning strategy:

1. Modifications of the Gufoni Maneuver

In the Gufoni maneuver, it is recommended to tilt the head back 45 degrees in the initial sitting position. The initial backward tilt helps move the otoliths away from the cupula, making repositioning easier.

The patient quickly lies down on the healthy side, maintaining this position for 30 s to 1–2 min, depending on the patient’s canal condition. The standard recommended time is 1–2 min, but 30 s is usually sufficient. Extended time may be more effective in patients with poor ducting.

When sitting up, it is advisable to lower the head 45 degrees. The forward tilt when sitting up aids in preventing the otoliths from entering the short arm of the posterior semicircular canal. It is advisable to maintain this position for 5 min to allow the otoliths that have returned to the utricle to adhere to the utricular macula.

2. For general HSC-BPPV

The Gufoni maneuver, when performed correctly, is effective for treating horizontal canal BPPV without the need for supine positions. This is especially beneficial for patients who have difficulty assuming a supine position. Bilateral manipulation is recommended to address otoliths in various positions within the canal.

3. For cupulolithiasis

Head-nodding movements are suggested to detach the cupulolith from the cupula before applying the Gufoni maneuver (19). This approach minimizes stimulation and helps in the effective repositioning of the otoliths (17).

## 5 Conclusion

Our virtual simulation study indicates that maneuvers avoiding supine position, especially the Gufoni maneuver, are more scientifically designed and safer. The Gufoni maneuver stands out for its versatility and effectiveness:

1. It can be performed bilaterally, allowing for the repositioning of otoliths in any location within the horizontal semicircular canal.
2. It has a shorter repositioning path compared to maneuvers involving supine position, potentially reducing patient discomfort.
3. It avoids the supine position, minimizing the risk of otoliths entering the semicircular canal from the utricle.

While other maneuvers like the 360-degree Lempert and Zuma maneuvers are also effective for otoliths in all positions, the Gufoni maneuver’s combination of effectiveness and patient comfort makes it a preferred choice in many cases. For cupulolithiasis, we recommend a “minimal stimulation strategy”: first using head-nodding movements to detach the cupulolith, then applying the Gufoni maneuver for repositioning. In performing the Gufoni maneuver, we recommend:

1. Tilting the head back 45 degrees in the initial sitting position.
2. Maintaining the healthy-side lying position for about 30 s.
3. When sitting up, lowering the head 45 degrees to prevent otoliths from entering the short arm of the posterior semicircular canal.

These maneuvers avoiding supine position can effectively reposition HSC-BPPV while reducing unnecessary discomfort for patients during the procedure. Future research should further validate these findings and explore the possibility of personalized repositioning strategies based on individual patient characteristics and specific otolith locations.

## Data Availability

The original contributions presented in the study are included in the article/Supplementary material, further inquiries can be directed to the corresponding author.
